# Quantitative Doppler-Echocardiographic Determination of Regurgitant Volume in Patients with Aortic Insufficiency

**DOI:** 10.2174/1874192400802010012

**Published:** 2008-03-04

**Authors:** Paul Schoenhagen, Ludwig Drude, Hermann H Klein, Mario J Garcia

**Affiliations:** a From the The Cleveland Clinic Foundation, Cleveland, Ohio, USA; b Kardiologische Gemeinschaftspraxis Marburg, Germany; c Klinikum Idar-Oberstein GmbH, Germany

**Keywords:** Aortic Insufficiency, Doppler-Echocardiography, Cardiac Catheterization, Regurgitant Volume.

## Abstract

**Background:**

The severity of aortic regurgitation (AR) can be determined by invasive or echocardiographic methods. We systematically compared quantitative invasive and echocardiographic data with semiquantitative invasive grades in a prospective series of patients.

**Methods:**

Using Doppler-echocardiography we determined the cardiac output over the aortic, pulmonary and mitral valve in 27 patients (20 with, 7 without AR). Aortic regurgitant volume was calculated as the difference between the cardiac output over aortic and pulmonary valve/ mitral valve. During angiography the severity of AR was assessed semiquantitatively by aortography and the regurgitant volume was calculated invasively as the difference between the left- and right ventricular cardiac output.

**Results:**

The echocardiographically and invasively determined regurgitant blood volume correlated closely (R≈0.8). The regurgitant volume increased with higher angiographic grade but there was significant overlap between adjoining qualitative grades.

**Conclusion:**

In patients with AR, quantitative echocardiographic and angiographic measurements of the regurgitant volume correlate closely.

## INTRODUCTION

The natural history of aortic regurgitation (AR) is characterized by an asymptomatic period of left ventricular compensation followed by decompensation with eventually irreversible left ventricular dysfunction. The management of patients with AR and in particular the timely indication of valve surgery is therefore dependent on a reliable estimation of the severity and progression of regurgitation and LV function [1]. The severity of AR has traditionally been estimated during cardiac catheterization by semiquantitative grading [2-4]. Quantitative echocardiographic methods have been developed to determine the hemodynamically active regurgitant volume noninvasively. The comparison between quantitative echocardiographic methods and angiographic grades has shown only modest correlation, which has been attributed at large to inaccuracy of the echocardiographic methods. We hypothesized that the discrepancy between echocardiography and angiography is due to the comparison of quantitative with qualitative data. We therefore systematically compared quantitative echocardiographic, quantitative invasive and semiquantitative invasive measurements in a prospective series of consecutive patients.

## METHODS

### Patients

Between July ‘90 and May ‘93 we examined 136 consecutive patients who were referred to the color imaging laboratory of the Philipps-University-Marburg for further evaluation of suspected valvular disease. All patients had undergone 2D and conventional Doppler studies before referral. Informed consent was obtained according to institutional guidelines. The study was approved by the Philipps-University Marburg. Fourty-four patients were excluded from the final analysis for the following reasons: no reliable visualization (n=2), pulmonary valvular disease or combined aortic valve disease (n=6), mitral valve disease (n=24), atrial septal defect (n=9), ventricular septal defect (n=3). Of the remaining 92 patients, only those 27 patients that were examined by cardiac catheterization and echocardiography within 24 hours were included in this study. Twenty of these patients had AR and 7 did not. The patients with AR had no concomitant, significant aortic stenosis (maximal systolic gradient of less than 30mmHg). The 7 patients in the control group had the following diseases: dilated cardiomyopathy (n=2), CAD (n=2), HTN (n=2), mitral valve prolapse without regurgitation (n=1).

### Cardiac catheterization:

#### General Procedure

##### Left Heart Catheterization

Access was established using the Seldinger technique from a femoral artery. After coronary angiography a 7F pigtail catheter was placed in the mid left ventricle. A left ventriculogram was recorded in the 30 degree RAO projection over several cardiac cycles. The catheter was then withdrawn into the ascending aorta for the aortography in a 30 degree RAO projection. For both ventriculography and aortography, 40 ml of contrast material were injected with a flow of 14 ml/sec.

### Semiquantitative Assessment of Aortic Regurgitation (Aortography)

The semiquantitative grading of AR was done according to established guidelines [2]. Grade 1 was defined as backflow without contrast of the complete LV, grade 2 as backflow with faint contrast of LV, grade 3 as dense contrast of LV same as aorta and grade 4 as contrast of LV more dense than aorta.

### Ventriculography

The images of the ventriculogram were analyzed off line. End-diastolic and end-systolic frames were chosen for planimetry. The pictures were projected from a projector (Tagano 35 CX) onto a calibrated screen. The heart base, the maximal length of the ventricular cavity and the left ventricular circumference were manually traced on the screen. The left ventricular stroke volume (LV) was calculated according to the area-length method [5,6]. The results of three consecutive cardiac cycles in sinus rhythm were averaged.

### Thermodilution

The right heart catheterization was performed *via *a femoral vein with a pulmonary artery flotation catheter. The tip of the catheter was positioned in the main stem of the right pulmonary artery. Through the proximal sidehole 10 ml 0.9% NaCl at room temperature were injected. At the thermistor at the distal end of the catheter the temperature were recorded.

The right ventricular (RV) cardiac output was calculated semiautomatically according to the Stewart Hamilton equation [7,8].

### Quantitative Assessment of Aortic Regurgitation

The invasive regurgitant volume (RVinvasive) was calculated as the difference between the left- and right-ventricular cardiac output (LVCO/RVCO) determined by ventriculography (LVCO) and thermodilution (RVCO).

### Echocardiography

#### General Procedure

All examinations were performed by an experienced physician sonographer, who was blinded to the results of the cardiac catheterization. The examinations were performed with a Sonotron Vingmed CFM750 machine, equipped with an imaging transducer of 3.5 or 2.75 Megahertz for the M-mode and 2D images. The echo-machine was connected online with a Macintosh Apple III personal computer equipped with an image processing software package. For off line analysis, the images were stored on optical disk and also printed on paper and recorded on a video recorder.

**Fig. (1):**
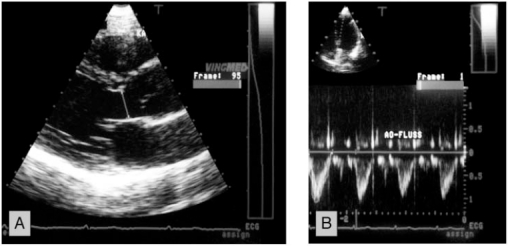
Example of the measurement of the flow area and the velocity time integral over the aortic valve (LVOT). Panel **A**: The area of the LVOT was calculated from a diameter in the parasternal long axis view. Panel **B**: The VTI in the LVOT was measured from the apical 4-chamber view with the sample volume in the LVOT.

**Fig. (2):**
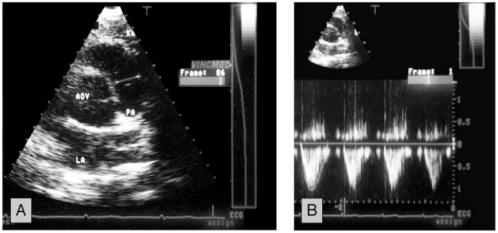
Example of the measurement of the flow area and the velocity time integral over the pulmonary valve. Panel **A**: The area of the pulmonary artery was calculated from a diameter measured at the level of the valve in the parasternal short axis. Panel **B**: The VTI in the pulmonary artery was measured from a parasternal short axis view with the sample volume at the level of the valve.

**Fig. (3):**
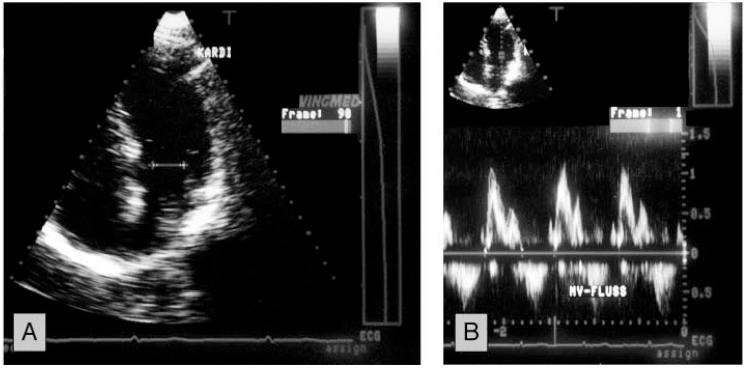
Example of the measurement of the flow area and the velocity time integral over the mitral valve. Panel **A**: The area of the mitral valve was measured from a diameter measured at the level between the mid and distal third of the valve leaflets in the apical 4-chamber view. Panel **B**: The VTI in the mitral valve was measured from the apical 4-chamber view.

The examination was performed in a left decubitus position. Complete 2D, conventional Doppler and color Doppler study with evaluation of all valves and ventricular function were performed according to ASE standards. The cross sectional areas (CSA) of the aortic, pulmonic and mitral valve were determined using the formula CSA = πr2 as follows: The maximal diameter of the left ventricular outflow tract (LVOT) was measured from a 2D-parasternal long axis view. The maximal diameter of the pulmonary artery (PA) was measured from a 2D-parasternal short axis view at the level of the pulmonic valve. The maximal diameter of the mitral valve (MV) was measured from a 2D apical 4-chamber view at the level between the mid and distal third of the valve leaflets (Figs. **1-3**).

The flow velocity was measured by pulsed Doppler. It was measured in a parasternal short axis view for the pulmonary valve with the sample volume directly distal of the valve. For the aortic valve it was measured from an apical 5-chamber view with the sample volume in the LVOT . For the mitral valve flow velocity was measured from a apical 4-chamber view with the sample volume at the level of the mitral valve, respectively (Fig. **1-3**).

### Quantitative Image Analysis

The Doppler flow signals of the aortic, mitral, and pulmonary valves were manually traced on the screen of the Echo machine and the velocity time integral (VTI) was calculated semiautomatically by planimetry. For each valve, 3 representative cycles that followed a sinus beat were analyzed, and the results averaged. The stroke volume was calculated according to the formula [9-13].

SV=VTI x A

(SV= stroke volume, VTI= velocity time integral, A= valve area)

The cardiac output (CO) was calculated by multiplying stroke volume (SV) and heart rate (HR):

CO=SV x HR

### Calculation of Regurgitant Volume (RVecho1, RVecho2)

The regurgitant volume (RVecho1) was calculated as the difference between the cardiac output of the aortic and pulmonary valve [14].

$$\text{RVechol}={\text{CO}}_{\text{LVOT}}-{\text{CO}}_{\text{PV}}$$

RVecho2 was calculated as the difference between the cardiac output of the aortic and mitral valve [15].

$$\text{RVecho2}={\text{CO}}_{\text{LVOT}}-{\text{CO}}_{\text{MV}}$$

### Statistical Analysis

For all measurements the mean, standard deviation and ranges are described.

Comparisons of the results were made by paired and unpaired t-test for paired and unpaired data, respectively. Simple linear regression analysis was employed to test the relation between two continuos parameters. Spearman rank correlation was used for the comparison of discrete angiographic grades and continuos parameters.

## RESULTS

### Patient Population

Mean age was 49.4±12.6 (23-69) years. Eighteen (66.7%) patients were male. Mean EF was 60.5±17.8%. LVEDd was 5.9±1.1 cm (4.1-7.7), LVESd was 4.0±1.01 cm (2.4-6.4). All patients were in normal sinus rhythm at the time of examination. The mean heart rate during catheterization was 73.9±14.4 bpm and during echocardiography 70.3±20.2 bpm.

### Echo

#### Cardiac Output Over the Aortic, Pulmonary and Mitral Valve

In the 20 patients with AR the cardiac output over the aortic, pulmonary, and mitral valve were: 9.4±3.2 l/min (5.5-17.0), 4.72±1.1 l/min (2.7-7.3), and 5.1±1.1 l/min (3.3-7.4), respectively.

In the 7 patients without AR the cardiac output over the aortic, pulmonary, and mitral valve were: 5.3±1.2 l/min (3.7-7.1), 5.1±1.1 l/min (3.5-7.3), and 5.1±1.4 l/min (3.3-7.8), respectively.

#### Regurgitant Volume

The calculated regurgitant volume in patients with AR was 4.7±2.7 (1.4-10.8) for the comparison of the aortic and pulmonary valve (RVecho1) and 4.3±2.6 (0.01-10.3) for the comparison of the aortic and mitral valve (RVecho2).

The calculated regurgitant volume in patients without AR was 0.2±0.8 (-0.8-1.6) for the comparison of the aortic and pulmonary valve (RVecho1) and 0.3±0.9 (-0.7-1.7) for the comparison of the aortic and mitral valve (RVecho2).

## INVASIVE

### Semiquantitative Grading of Aortic Regurgitation

Semiquantitative grading of aortic regurgitation by aortography showed grade 0 in 7 patients (26%), grade 1 in 1 patients (4%), grade 2 in 8 patients (30%), grade 3 in 9 patients (33%) and grade 4 in 2 patients (7%).

#### Cardiac Output as Determined by Thermodilution and Ventriculography.

In the 20 patients with AR the right-and left-ventricular cardiac output were 5.9±1.3 l/min (3.6-9.0) and 10.7±3.8 l/min (6.0-19.8), respectively.

In the 7 patients without AR the right-and left-ventricular cardiac output were 5.0±0.9 l/min (3.5-6.6) and 5.1±1.7 l/min (2.7-7.3), respectively.

### Regurgitant Volume

In the 20 patients with AR the calculated regurgitant volume was 4.7±3.3 l/min (0.81-12.8).

In the 7 patients without AR the calculated regurgitant volume was 0.06±1.1 l/min (-1.9-1.3).

Comparison between Echocardiographic and Invasive Method

#### Comparison of RVecho1 to RVecho2 (Fig. 4)

The regurgitant volume calculated from aortic and pulmonary valve (RVecho1) was compared to the regurgitant volume calculated from aortic and mitral valve (RVecho2). For the entire group, the correlation was 0.98; p<0.0001; y= 0.93x-0.011; SE= 0.038.

#### Comparison of RVinvasive to RVecho (Fig. 5)

The echocardiographic regurgitant volume RVecho1 (aortic/pulmonary valves) and RVecho2 (aortic/mitral valves) were compared with the angiographic regurgitant volume (RVinvasive). For RVecho1 the correlation was 0.8; p<0.0001; y=0.69x+1.07; SE= 0.10.

For RVecho2 the correlation was 0.78; p<0.0001. y=0.64x+0.99; SE=0.10.

#### Comparison of RV to Angiographic Grades (Fig. 6 and 7)

Table **1** shows the comparison of the RV calculated by echocardiographic and invasive method and the angiographic grades. There was a significant correlation between higher grades of severity and increased RVecho 1 (p=0.004, rho=0.70), RVecho2 (p=0.005, rho=0.68), and RVinvasive (p=0.002, rho=0.73; Spearman Rank Correlation).

However, because of a wide range of the regurgitant volume in each grade, no significant differences of the echocardiographic RV was found between angiographic grades 2 and 3. (RVecho1: 3.4+/-1.8 vs. 5.3+/-2.8 l/min; p=0.12, RVecho2: 3.03+/-1.7 vs. 4.97+/-2.6 l/min; p=0.10, RVinvasive: 3.8+/-2.9 vs. 4.68+/-3.1 l/min; p=0.56)

## DISCUSSION

In this study we systematically compared quantitative echocardiographic, quantitative invasive and semiquantitative invasive data determining the severity of aortic insufficiency. We found a high correlation (R=0.98) between two quantitative echocardiographic methods quantifying the regurgitant volume over the aortic valve (RVecho1 and RVecho2). Although the correlation between angiographic and echocardiographic quantitative methods was good, the correlation between semiquantitative angiographic grades and RV was only moderate and semiquantitative grading could not differentiate grade 2 and 3 because of significant overlap between grades.

These results have important implications for the approach to patients with AR. The angiographic semiquantitative classification has been the historical “gold standard” for estimating severity of AR and grade 3 has been used as a cutoff point above which to consider valve surgery. Our results suggest that angiographic grades 2 and 3 do not reliably differentiate groups with different hemodynamically active regurgitant volume. This is consistent with results of previous studies. Kitabatake examined 10 patients with isolated AR, 13 with additional valve disease and 10 control patients [14]. Using duplex Doppler echocardiography the aortic regurgitant fraction (RF) was calculated from systolic aortic and pulmonary flow. A fair correlation was found between RF_Echo_ and semiquantitative grades (r=0.80, p<0.01), with significant overlap between grades. Rokey *et al*. examined 6 patients with isolated AR and 19 patients with isolated MR [15]. Mitral and aortic valve flow were obtained with 2-dimensional doppler and RF was calculated. Results were compared with measurements of RF obtained by combined LV angiography and thermodilution. No significant correlation was found between RF by either method and angiographic grades. Kurokawa examined 15 patients with AR, 17 patients with MR and 41 normal controls [15]. The authors measured cardiac output over the mitral and aortic valve respectively. RV and RF was calculated. The comparison with angiographic grading showed significant overlap between adjacent grades.

These and other studies [17] describe significant overlap of the regurgitant volume between angiographic grades. This overlap is caused by the fact that the angiographic grades comprise a continuum of values of regurgitant volume without a sharp border between grades. Also other factors such as location of the catheter, size of the aorta and LV, and aortic blood pressure may influence the degree of LV opacification during aortography.

Previous studies generally report a good correlation between echocardiographic, szintigraphic and invasive quantitative methods determining RV: Kitabatake calculated the regurgitant fraction (RF) invasively from right ventricular stroke volume, determined by thermodilution and left ventricular stroke volume determined by ventriculography [14]. A close correlation was found between the RF estimated with Doppler and catheter technique in patients with isolated AR (r=0.96, p<0.01). Rokey compared Doppler-echocardiography with measurements of RF obtained by combined LV angiography and thermodilution [16]. A significant correlation was observed between the two methods (r=0.91, SEE = 7%). Zhang examined 26 patients with AR and 23 normal controls by combined 2-dimensional and Doppler-echocardiography and calculated RF by comparing transmitral volume and left-ventricular stroke volume [17]. The RF was also calculated by radionuclide ventriculography. The comparison between echocardiographic and invasively calculated RF was good. Kurokawa calculated RF by radionuclide angiography [15]. The RF calculated by echocardiographic and invasive measurements correlated well (r = 0.79 p < 0.01; n = 11).

In calculating the cardiac output over individual valves by multiplying the velocity time integral (VTI) and the outflow tract area several technical limitations have to be considered: The calculation of the outflow tract area from one measured diameter assumes a circular shape during systole. Several studies have shown a nearly circular anatomy of the aortic and pulmonary valves and a constant valve area during systole [18-20]. On the other hand the mitral valve has been shown to have an oval anatomy with variable valve area during systole secondary to the biphasic flow pattern in diastole [21,22]. Therefore the mitral valve orifice has often been calculated by oval formulas or determined by planimetry [23-25]. However, several studies have reported good results with calculation based on circular formula [26,27]. We found a high correlation between the two echocardiographic methods to calculate the regurgitant volume confirming the usefulness of a circular formula to calculate the cross-sectional area of the mitral valve.

The goal of our study was to compare noninvasive, echocardiographic quantification of aortic insufficiency versus quantitative and qualitative invasive methods. We did not perform a comparison of the described echocardiographic quantitative method to a comprehensive echocardiographic evaluation as performed typically in clinical practice. This typically includes flow mapping to determines the spatial extension of the regurgitant jet [28-31], the determination of the jet area at the valve level (vena contracta) [32], directional flow in the ascending /descending aorta [33], pressure-half-time [34,35] and the proximal isovelocity surface area (PISA) method [36,37].

However, as quantification of flow events has more recently been described using three dimensional echocardiography [38,39] and magnetic resonance imaging [40-42], routine quantification of the regurgitant volume in patients with aortic insufficiency may become possible and the described method can serve as a reference.

### Limitations

The small number of patients is a major limitation of our study. Also, the regurgitant volume is dependent on hemodynamic conditions. Therefore the time delay between echocardiographic and invasive examination may have affected the correlation between echocardiography and angiographic grades. In order to minimize that influence we included only patients who had the two examinations within 24 hours. The results of the RV calculated in patients without AR show a relative large range with both echocardiographic and invasive calculation. A careful comparison of *qualitative* results obtained with 2D Echo and *quantitative *echocardiographic results and clinical findings is therefore necessary.

## CONCLUSION

The results of our study show a good correlation between echocardiographic and invasive quantitative calculations of the regurgitant volume in patients with aortic insufficiency. The comparison to the semiquantitative angiographic grades show that grades 2 and 3 do not reliably differentiate groups with significant different regurgitant volume. Although angiographic grading of severity is considered the traditional “gold standard”, management decisions in patients with aortic regurgitation should therefore not rely on angiographic grades alone but incorporate a quantitative assessment of the hemodynamically active regurgitant volume.

## Figures and Tables

**Fig. (4):**
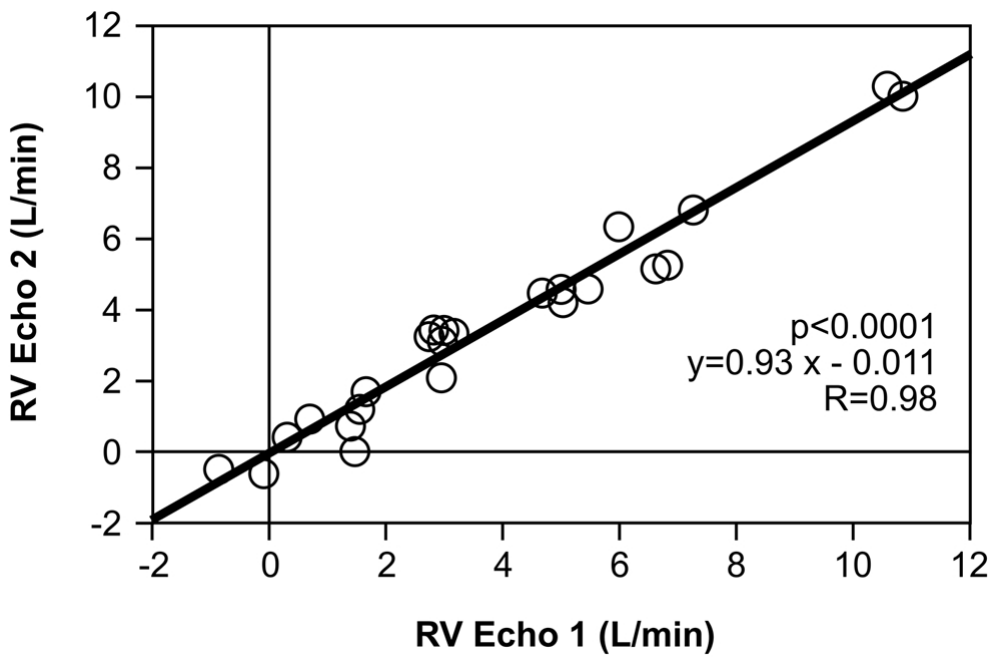
Comparison of the echocardiographic regurgitant volume measured by comparison of the aortic and pulmonary valve (RVecho1) and aortic and mitral valve (RVecho2).

**Fig. (5):**
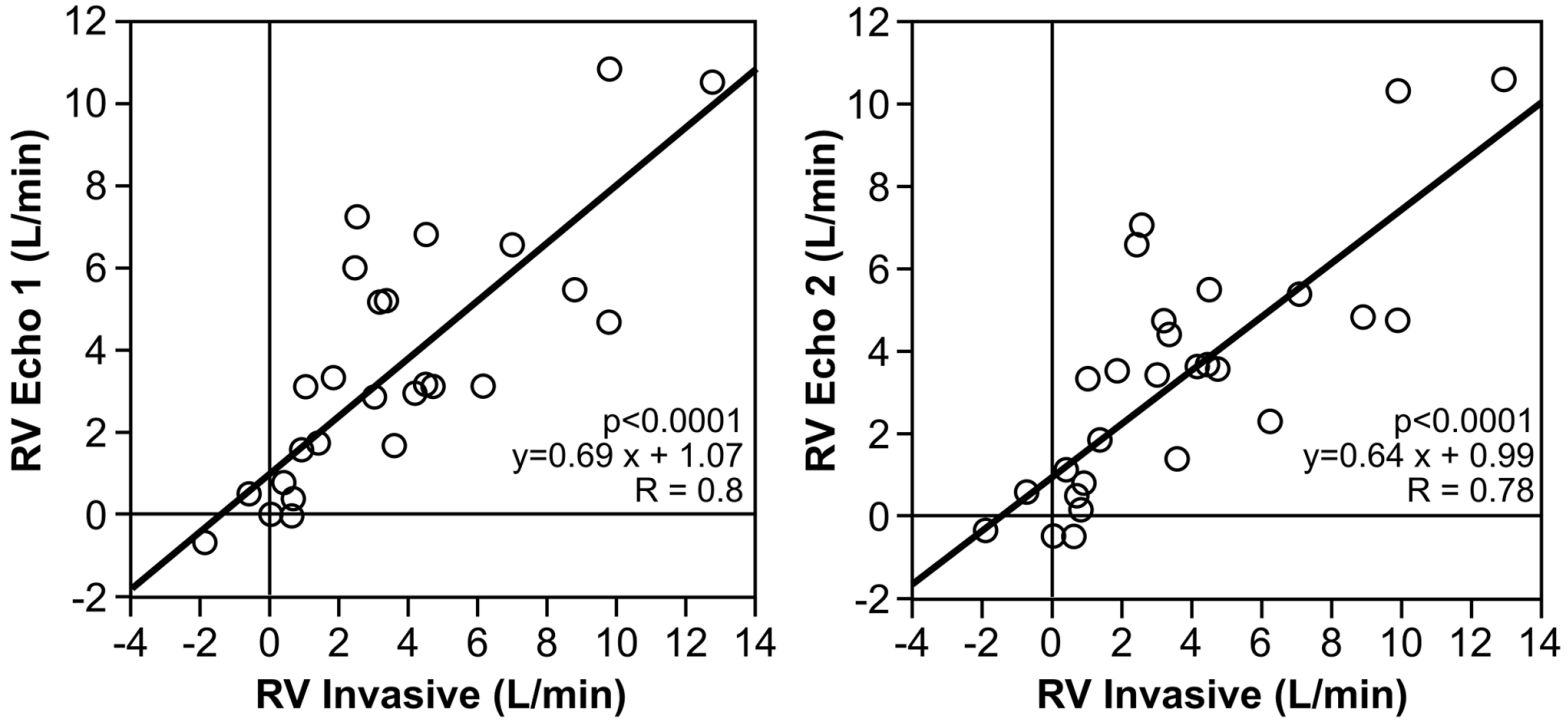
Comparison of the echocardiographic regurgitant volume (RVecho1 and RVecho2) with the invasive measured RV.

**Fig. (6):**
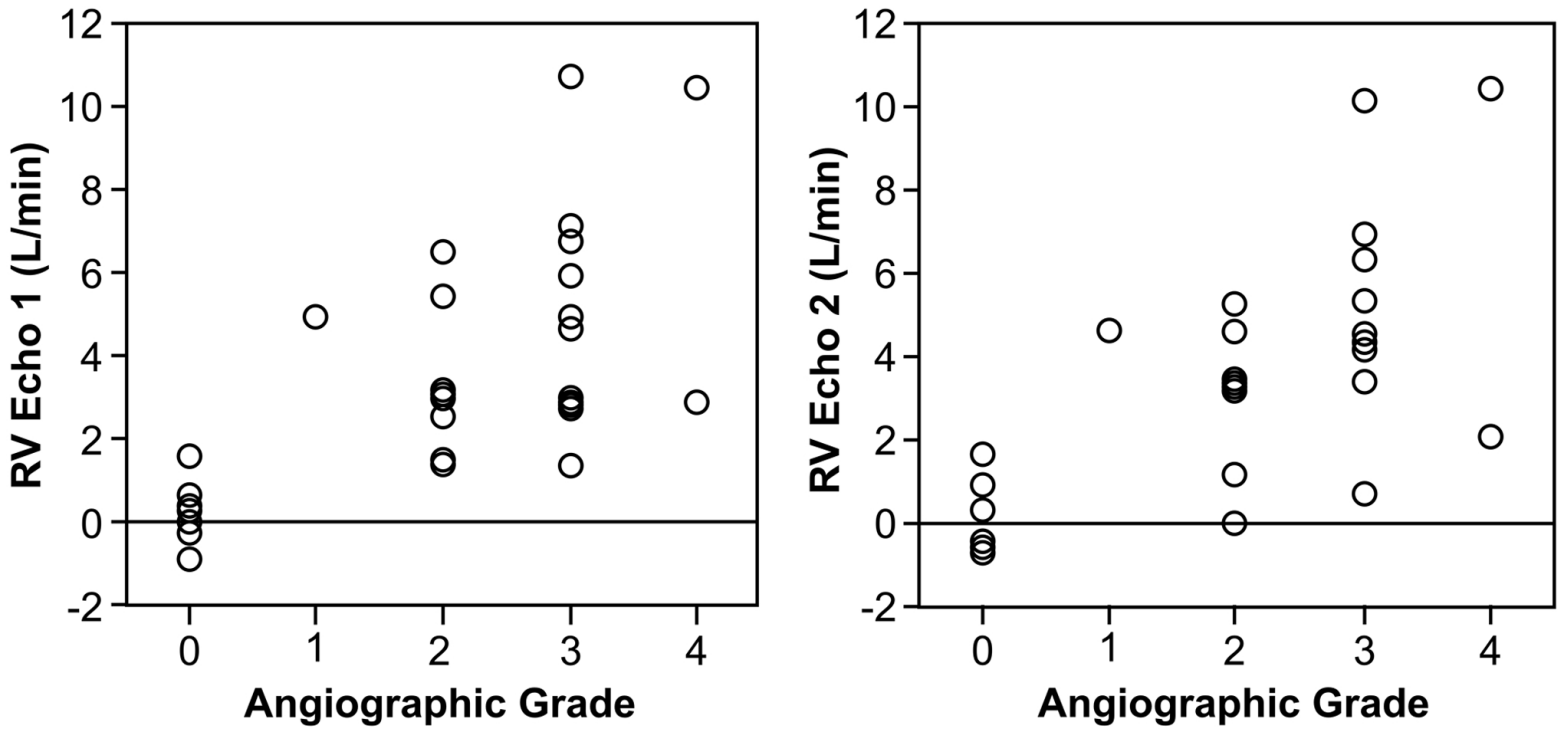
Comparison of the echocardiographic regurgitant volume (RVecho1 and RVecho2) with the angiographic grades.

**Fig. (7):**
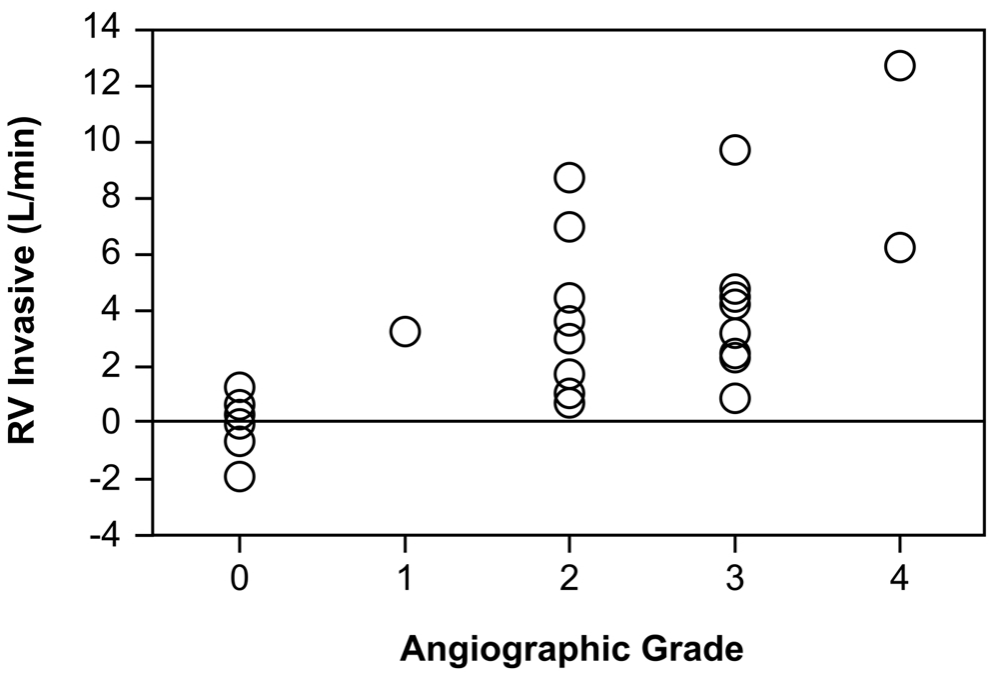
Comparison of the invasive regurgitant volume (RVinvasive) with the angiographic grades.

**Table 1.** Comparison of Echocardiographic and Invasive determined Regurgitant Volume

	RVecho1	RVecho2	RVinvasive
**Grade 0** (n=7)	0.03+/-0.15	0.04+/-0.15	-0.06+/-0.3
**Grade 1** (n=1)	4.97	4.62	3.2
**Grade 2** (n=8)	3.4+/-1.8	3.03+/-1.7	3.8+/-2.9
**Grade 3** (n=9)	5.30+/-2.8	4.97+/-2.6	4.68+/-3.1
**Grade 4** (n=2)	6.76	6.22	9.5

RVecho1 = echocardographically determined regurgitant volume (comparison aortic and pulmonary valve). RVecho2 = echocardographically determined regurgitant volume (comparison aortic and mitral valve). Rvinvasive = invasively determined regurgitant volume.

## ABBREVIATIONS

AR =: Aortic Regurgitation
CAD =: Coronary Artery Disease
CSA =: Cross Sectional Area
